# P041. Analysis of body mass index, psychiatric comorbidity, sleep-wake pattern and occurrence of fatigue in episodic and chronic migraine patients

**DOI:** 10.1186/1129-2377-16-S1-A188

**Published:** 2015-09-28

**Authors:** Cinzia Lucchesi, Filippo Baldacci, Martina Cafalli, Elisa Dini, Gabriele Siciliano, Ubaldo Bonuccelli, Sara Gori

**Affiliations:** Department of Clinical and Experimental Medicine, Neurology Unit, University of Pisa, Pisa, Italy

## Background

Migraine clinical picture and life-time disease course can be highly heterogeneous, with a subgroup of patients developing chronic migraine, a highly disabling condition, associated with high socio-economic burden. Moreover, migraine clinical spectrum is expanded by the association with different comorbid/coexisting conditions and interictal dysfunctions, contributing to modulate migraine clinical profile. Taking this scenario into consideration, the aim of this study was to systematically evaluate migraine clinical features, body mass index (BMI), depressive and anxiety symptoms, sleep-wake pattern and occurrence of fatigue in a sample of episodic and chronic migraine patients, as well as their reciprocal interaction.

## Methods

One hundred and fifty patients with a diagnosis of migraine without aura were enrolled; 75 patients fulfilled criteria for episodic migraine and 75 for chronic migraine (ICHD-3 beta). Patients with comorbid/coexisting conditions or in treatment with migraine preventive drugs were excluded. Data regarding age, gender, monthly frequency of migraine attacks, disease duration and BMI were collected. Migraine-related disability, presence of anxiety and depressive symptoms, subjective sleep quality, chronotype, fatigue and daily sleepiness were, respectively, evaluated using the following questionnaires: Migraine Disability Assessment Score (MIDAS), Generalized Anxiety Disorder 7-item scale (GAD-7), Patient Health Questionnaire 9-item scale (PHQ-9), Pittsburgh Sleep Quality Index (PSQI), reduced Morningness-Eveningness Questionnaire (rMEQ), Fatigue Severity Scale (FSS), Epworth Sleepiness Scale (ESS).

## Results

Mean age (p = 0.012), disease duration (p < 0.001), BMI score (p = 0.012) and MIDAS score (p = 0.005) were significantly higher in chronic compared to episodic migraineurs; furthermore, mean GAD-7 score (p = 0.019), PHQ-9 score (p < 0.001), PSQI score (p = 0.015) and FSS score (p < 0.001) were higher in chronic migraine patients. No statistically significant differences were documented in gender distribution, mean ESS and rMEQ scores between episodic and chronic migraineurs. A correlation analysis (Rho coefficient of Spearman) carried out in the total sample of 150 migraineurs, documented a statistically significant, positive correlation between monthly frequency of migraine attacks and patients’ age (p < 0.001), disease duration (p <.0.001), BMI score (Rho 0.177, p = 0.049), MIDAS score (p < 0.001), GAD-7 score (p = 0.019), PHQ-9 score (p < 0.001), PSQI score (p = 0.006) and FSS score (p < 0.001).

## Discussion

Data from the present report seem to expand the concept of migraine as a continuum or spectrum, with higher BMI score and greater occurrence of anxiety-depressive symptoms, poor sleep quality and fatigue in chronic migraine patients compared to episodic migraineurs; further investigation is certainly necessary in order to better define the biological basis and mechanisms associated with migraine transformation from episodic to chronic pattern.

Written informed consent to publish was obtained from the patient(s).

